# Imperatorin exerts antioxidant effects in vascular dementia via the Nrf2 signaling pathway

**DOI:** 10.1038/s41598-022-21298-x

**Published:** 2023-04-05

**Authors:** Xiangping Liao, Ziliang Zhang, Min Ming, Shanquan Zhong, Jianping Chen, Ying Huang

**Affiliations:** 1 Department of Psychology, The Third People’s Hospital of Ganzhou City, Ganzhou, 341000 Jiangxi China; 2 Department of Neurology, Xinfeng County People’s Hospital, Ganzhou, 341000 Jiangxi China; 3 Department of Neurology, The First Affiliated Hospital of Gannan Medical University, GANZHOU, China; 4 Ganzhou People’s Hospital, Ganzhou, 341000 Jiangxi China; 5 Key Laboratory of Prevention and Treatment of Cardiovascular and Cerebrovascular Diseases, Ministry of Education, Gannan Medical University, Ganzhou, 341000 Jiangxi China; 6 Gannan Branch Center of National Geriatric Disease Clinical Medical Research Center, Gannan Medical University, Ganzhou, 341000 Jiangxi China

**Keywords:** Drug discovery, Neuroscience

## Abstract

Imperatorin, an active ingredient extracted from Angelica and Qianghuo, has anti-inflammatory, anti-oxidative stress damage, blocking calcium channels, and other properties. Our preliminary findings revealed the protective role of imperatorin in the treatment of vascular dementia, we further explored the underlying mechanisms concerning the neuroprotection function of imperatorin in vascular dementia. The cobalt chloride (C_O_Cl2)-induced chemical hypoxia and hypoglycemia of hippocampal neuronal cells was applied as in vitro vascular dementia model. Primary neuronal cells was isolated from the hippocampal tissue of SD suckling rats within 24 h of birth. Hippocampal neurons were identified by immunofluorescence staining of microtubule-associated protein 2. Silencing or overexpression of Nrf2 was conducted by transfection of corresponding plasmids in hippocampal neuronal cells. Cell viability was detected by MTT assay to determine the optimal modeling concentration of CoCl2. Mitochondrial membrane potential, intracellular reactive oxygen species and apoptosis rate was measured by flow cytometry. The expression of anti-oxidative proteins was detected by quantitative real-time PCR and western blot, including Nrf2, NQO-1 and HO-1. Nrf2 nuclear translocation was detected using laser confocal microscopy. The modeling concentration of CoCl2 was 150umol/l, and the best interventional concentration of imperatorin was 7.5umol/l. Significantly, imperatorin facilitated the nuclear localization of Nrf2, promoted the expressions of Nrf2, NQO-1, and HO-1 relative to the model-control group. Moreover, imperatorin reduced the mitochondrial membrane potential and ameliorated CoCl2-induced hypoxic apoptosis in hippocampal neurons. On the contrary, silencing Nrf2 completely abrogated the protective effects of imperatorin. Imperatorin might be an effective drug for preventing and treating vascular dementia.

## Introduction

Vascular dementia (VD) is recognized as the only preventable dementia that might be reversed by interventions within the early phase^1^, with complex pathophysiological mechanisms, including oxidative stress injury, apoptosis, autophagy, inflammation and synaptic plasticity damage, etc^2,3^. And that chronic cerebral hypoperfusion (CCH) is known to play an important role in the pathogenesis of VD^4^. Moreover, CCH usually resulted in oxidative stress damage in brain tissue^5^. Therefore, pathophysiologic and mechanistic understanding of CCH-induced VD is of great practical significance and application value and may contribute to produce promising treatment approaches.

Studies have confirmed that the nuclear factor E2-related factor 2 (Nrf2)/antioxidant response element (ARE) signaling pathway has a vital role in antagonizing chronic cerebral hypoperfusion injury^6^. Oxidative stress oxidizes the active cysteine residues of Kelch-like-ECH associated protein 1 (Keap1), leading to its inactivation and dissociation from Nrf2 protein^7,8^. Nrf2 remains stable and transfers into the nucleus, where it combines with smaf of the nucleus to form the Nrf2-Smaf complex, and binds with ARE in a specific sequence^9^. It not only increased the expression of downstream heme oxidase-1 (HO-1) and phosphate adenine dinucleotide quinone oxidoreductase-1 (NQO1) [Buendia et al.,2016], but also further promoted the expression of antioxidant enzymes and detoxification enzymes, such as total superoxide dismutase (SOD) and glutathione peroxidase (GSH-PX), which neutralizing excess reactive oxygen species (ROS), maintaining the redox reaction and protecting brain cells^10,11^.

Imperatorin (IMP) [9-[(3-methylbut-2-en-1-yl)oxy]-7H-furo[3,2-g]chromen-7-one] is a purely natural active furanocoumarin, one of the effective ingredients in the traditional Chinese medicines Cnidium Monnieri, Angelica Dahurica and Qianghuo, of Umbelliferae family. Modern pharmacological studies have confirmed that IMP has anti-oxidative, anti-inflammatory, anti-apoptosis, anti-thrombosis, inhibiting cholinease activity, blocking calcium channels, anti-convulsions, etc^12–14^.

Some researchers reported^15^ IMP can activate Nrf2/ARE signaling pathway. Wang et al. found that IMP can reverse the expression of apoptosis genes Bcl-2 and Bax through the mitochondrial pathway, and reduce the apoptosis of oxygen–glucose deprivation reperfusion cell model^16^. It has also been found that IMP can ameliorate lipopolysaccharide-induced learning and memory impairment by decreasing pro-inflammatory cytokines and regulating brain-derived neurotrophic factors^17^. In addition, our previous animal experiments showed that IMP can improve cognition in vascular dementia rats by inhibiting apoptosis and alleviating the damage of hippocampal synaptic plasticity^18^. Based on previous animal experiments, we hypothesized in this research whether IMP has anti-oxidative effect on the VD cell model of hippocampal neuronal injury induced by hypoxia and hypoglycemia and the possible mechanism of regulating the Nrf2 signal pathway.

## Materials and methods

### Animals and neuronal cell

All experiments were performed in accordance with the ethical guidelines and regulations of the Institutional Animal Care and Use Committee of the First Affilated Hospital of Gannan Medical University (Ganzhou, China). And the study was carried out in compliance with the ARRIVE guidelines. Hippocampal tissues of Spruage-Dawley Suckling rats within 24 h of birth were used as the source of hippocampal neurons. SPF Spruage-Dawley rats within 24 h of birth were provided by the Experimental Animal Center of Gannan Medical University [License No. : SCXK(GAN) 2014-0001].

### Primary cell culture and identification of hippocampal neuronal cells

After hippocampi were dissected and dissociated briefly, the dissociated cells were maintained in the chemically conditioned Neurobasal/B27/Glutamax medium. On the third day, an appropriate amount of 10 mM cytarabine was added to the culture and incubated for 24 h to inhibit the growth of other cells. And then the cytarabine solution was discarded, and neurobasal medium was still used to maintain the culture for 4 days before use. After 7 days of culture, the purity of cultured hippocampal neuronal cells was identified by immunofluorescence staining of 4',6-diamidino-2-phenylindole (DAPI) and microtubule-associated protein 2 (MAP2). Neuronal purity = MAP2 (+)/DAPI (+)×100%.

### Established Model of CoCl2-induced chemical hypoxia and hypoglycemia

We used CoCl_2_·6H_2_O (purity ≥ 98%, Sigma) to established model of CoCl2-induced chemical hypoxia and hypoglycemia, CoCl2-6H_2_O was prepared at a concentration of 50 mmol/l by dissolving 0.6 g in 50 ml of sterile double-distilled water.

Different concentrations of CoCl_2_ (50 μmol/l, 100 μmol/l, 150 μmol/l, 200 μmol/l, 300 μmol/l, 400 μmol/l) were prepared by diluting with sugar-free DMEM medium to dilute and incubated at 37 °C and 5% CO_2_ for 24 h. Cell viability was determined by MTT kit (Abcam Cambridge, UK). Calculated cell viability according to the following formula: viability (%) = OD (measured value) − OD (blank value)/OD (control value) − OD (blank value) × 100%. Half of the maximum inhibitory concentration of CoCl2 was designated as the optimal condition for simulating ischemia and hypoxia in hippocampal neuronal cells.

### Screened the concentration of IMP on CoCl2-induced hippocampal neuronal cells

The hippocampal neuronal cells were inoculated into 96-well plates at 5 × 10^4^ cells/well and incubated with different concentrations of IMP (2.5 μmol/l, 5.0 μmol/l, 7.5 μmol/l, 10 μmol/l, 12.5 μmol/l) on the sixth day for 24 h incubation. Then the neuronal cells were divided into normal and CoCl2-control group. CoCl2 group was incubated with CoCl2 solution for 24 h. The cell viability was also detected by MTT assay.

### Measurement of apoptosis rate

Apoptosis was detected by using flow cytometer with the Annexin V-FITC double staining method. After aspirating and discarding the cutlture, neuronal cells were added 0.25% trypsin without EDTA for 6 min, following with added 20% fetal bovine serum about 3 min. The cells were collected, centrifuged at 1000 g for 5 min, and then resuspended in PBS. 100,000 resuspended cells were centrifuged at 800 g for 5 min, and 195 μl FITC binding solution was added to resuspend the cells. Next, added 5 μl FITC and 10 μl PE, and mixed gently. After incubation for 20 min under protection from light, the cells were placed in ice boxes and set on the flow cytometer within 1 h. FlowJo analyzed the data, and membrane protein V-PI stained cells were differentiated into healthy cells (membrane protein V-/PE-), early apoptotic cells (membrane protein V + /PE-) and late necrotic cells (Annexin V + /PE +). In this study, the percentage of apoptotic cells was calculated by the sum of the percentages of early and late apoptotic cells.

### Measurement of mitochondrial membrane potential

Mitochondrial membrane potential of hippocampal neuronal cells was detected using flow cytometer and JC-10 kit (Biosharp, China) according to the manufacturer, instructions. When the mitochondrial membrane potential was high, JC-10 aggregated in the matrix of mitochondria and formed polymer, which could produce red fluorescence. When the mitochondrial membrane potential was low, JC-10 could not aggregate in the matrix of mitochondria, resulting in green fluorescence. The ratio of red to green fluorescence was used to measure the mitochondrial membrane potential.

### Measurement of Nrf2 nuclear translocation

In this experiment, disposable cell slides were used. Prior to the experiment, cell slides were placed in a 24-well plate, and routinely coated with PDL overnight. Inoculated in a 24-well plate at a density of 4×10^5^ cells/ml for 6 days, after different concentrations of IMP were added for 24 h. The IMP solution was discarded, and then CoCl2 was added and incubated for 24 h. After aspirating and discarding the medium, washed 3 times with PBS, 4% paraformaldehyde was added and fixed at room temperature for 30 min, and paraformaldehyde was aspirated. After washing with PBS, a blocking solution consisting of 5% goat serum, 0.3% triton-100 were added for 30 min. Then rabbit-anti-NQO-1 (diluted at 1: 200, Abcam Cambridge, UK) was used and incubated overnight at 4 °C in the refrigerator and washed with PBS. Next, Cy3 labeled goat anti-rabbit IgG (H+L) was added and diluted with a special fluorescent secondary antibody diluention of 1:500. After incubating for 1 h, washed with PBS, and finally added 10 μg/ml DAPI and incubated for 5 min at room temperature away from light. After washing, removed the cell slides, added an appropriate amount of anti-fluorescence quencher, and placed on the cover glasses, observed and took pictures.

### Measurement of ROS

ROS was detected using flow cytometer. Dichlorofluorescin diacetate (DCFH-DA) was diluted with serum-free medium at 1:1000, which leading to a final concentration of 10 μM. Cell culture medium was discarded, DCFH-DA was added, and then incubated at 37 °C for 20 min. After washing three times with serum-free medium, the cells were washed with 1 ml PBS and centrifuged at 1500 RPM for 5 min. Next, the supernatant was discarded and 300 μl PBS was added to resuspend the cells. Finally, flow cytometry was used for detection.

### Buliding of carrier

According to the gene sequence of Nrf2, three siRNA primers were designed, as shown in the Table 1.

**Table 1:** Three gene sequences of Nrf2.

Title of siRNA	Sequence of siRNA
Nrf2 (house mouse)siRNA-1-333	AGACAUAGAUCUUGGAGUATT UACUCCAAGAUCUAUGUCUTT
Nrf2 (house mouse)siRNA-2-2021	GCAAGAAGCCAGAUACAAATT UUUGUAUCUGGCUUCUUGCTT
Nrf2 (house mouse)siRNA-3-890	UGACAGAAAUGGACAGCAATT UUGCUGUCCAUUUCUGUCATT

### Validation and detection of fluorescence quantitative PCR

Total RNA from hippocampal neuronal cells was extracted with Trizol (Invitrogen, USA). cDNA was reversed using a reverse transcription kit (PrimeScript™, JPN). PCR reactions were then tested using a 25 μl reaction system (CFX96 Real-Time PCR Detection System, Bio-Rad). Cycling conditions were performed as followings: 30 s at 95 °C, 30 s at 60 °C, 40 PCR cycles (95 °C, 5 s, 60 °C, 30 s), 95 °C, 10 s, 65 °C, 5 s; 95 °C, 5 s. Melt curve analysis was performed to verify primer specificity. Data was displayed as a fold change above the proliferative condition mRNA levels using 2^−∆∆Ct^ values. Primer design and synthesis of target genes Nrf2, HO-1, NQO-1 and β-Actin sequences were derived from the database (https://pubmed.ncbi.nlm.nih.gov/), and primer design and synthesis were carried out by Shenggong (Shanghai, China), see details in Table 2.

**Table 2:** Primer sequences.

Primer	Sequences
Nrf2	Forward: 5’-CATTTGTAGATGACCATGAGTCGC-3’
	Reverse: 5’-GCCAAACTTGCTCCATGTCC-3’
HO-1	Forward: 5’-TCTGCAGGGGAGAATCTTGC-3’
	Reverse: 5’-TTGGTGAGGGAAATGTGCCA-3’
NQO-1	Forward: 5’-GGCCATCATTTGGGCAAGTC-3’
	Reverse: 5’-TCCTTGTGGAACAAAGGCGA-3’
β-actin	Forward: 5’-TGGAGCAAAGATCCCCCAAA-3’
	Reverse: 5’-TGCCGTGGATACTTGGAGTG-3’

Nrf2 interference vector and overexpression vector were constructed and transfected into target cells respectively. Fluorescence quantitative PCR was used to verify the transfection efficiency. The relative expression levels of Nrf2, HO-1 and NQO1 were calculated using β-actin as an internal reference. All primers were designed by Primer 5.0 software and NCBI Primer Blast, synthesized by General Biological Systems (Anhui, China), and purified by PAGE, see details in Table 3.

**Table 3:** Primers sequences.

Title of primer	Sequence of primer	Length (bp)	Annealing temperature (℃)
Nrf2 F	ACGGAAAACAAGCAGCAGG	222	59.2
Nrf2 R	GGTGGGATTTGAGTCTAAGGAGT		
HO-1 F	AGGTCCTGAAGAAGATTGCG	279	58.7
HO-1 R	GGCGAAGAAACTCTGTCTGTGA		
NQO1F	GGCCAATTCAGAGTGGCAT	163	58.2
NQO1 R	GCAAAGTAGAGTGGTGACTCC		
β-actin F	GCCATGTACGTAGCCATCCA	375	59.5
β-actin R	GAACCGCTCATTGCCGATAG		

### Measurement of oxidative levels of protein by western blot

Protein concentrations were determined with the BCA Protein Assay Kit (Thermo Fisher Scientific, United States).The sample protein 25 μg was added into lysate to denaturate, and then electrophoresis by SDS-PAGE, transferred to PVDF membranes. After being blocked with 5% skimmed milk for 1 h, the membranes were incubated with primary antibodies overnight on a shaker. The primary antibodies (Abcam, Cambridge, UK), including rabbit-anti-Nrf2 (diluted at 1: 1000), rabbit-anti-HO-1 (diluted at 1: 1500), rabbit-anti-NQO-1 (diluted at 1: 1500), rabbit-anti-β-actin, and rabbit-anti-GAPDH (diluted at 1:3000), were used as a loading control. After being washed with Tris-bufered saline, the membranes were incubated with horseradish peroxidase conjugated anti-rabbit secondary antibody (diluted at 1:1500, Abcam, Cambridge, UK). Finally, Image J software (NIH, Bethesda, MD, USA) was used to analyze the band density of western blots.

### SiRNA and overexpression plasmid transfection

Transfection of Nrf2 siRNA and overexpression plasmid were performed according to the manufacturer’s instructions. Cells were seeded into 96-or 6-well culture plates and transfected with the siRNA and overexpression plasmid duplexes at approximately 70–80% confluence with EntransterTM-R4000 transfection reagent according to the manufacturer’s instructions. Cells transfected with the control siRNA were treated in parallel. The cellular levels of siRNA and overexpression plasmid-specific proteins were detected by western blot and q-PCR. All experiments were performed after a 24 h transfection.

### Statistics

All analyses were done using SPSS 22.0 software (SPSS Inc., Chicago, USA, https://www.onlinedown.net/soft/577760.htm), and GraphPad Prism 8.0 (GraphPad Software Inc., San Diego, CA, USA, http://www.downxia.com/downinfo/260957.html) was used for graphing. Quantitative data were expressed as mean ± standard deviation (mean ± SD), and P < 0.05 was statistically significant. Normality of the data distribution was assessed using the Shapiro–Wilk test. Cell viability, apoptosis rate of hippocampal neuronal cells, the relative expression of Nrf2, NQO-1, HO-1, β-actin mRNA and proteins among different groups were analyzed using one-way analysis of variance (ANOVA) followed by SNK post hoc analysis. MannWhitney U test was used for non-normally distributed data.

### Ethical approval

All animal study was reviewed and approved by the Institutional Animal Care and Use Committee of the First Affilated Hospital of Gannan Medical University (Gannan Medical University, Ganzhou, China).

## Results

### Identification of the purity of hippocampal neuronal cells

**Figure 1:**
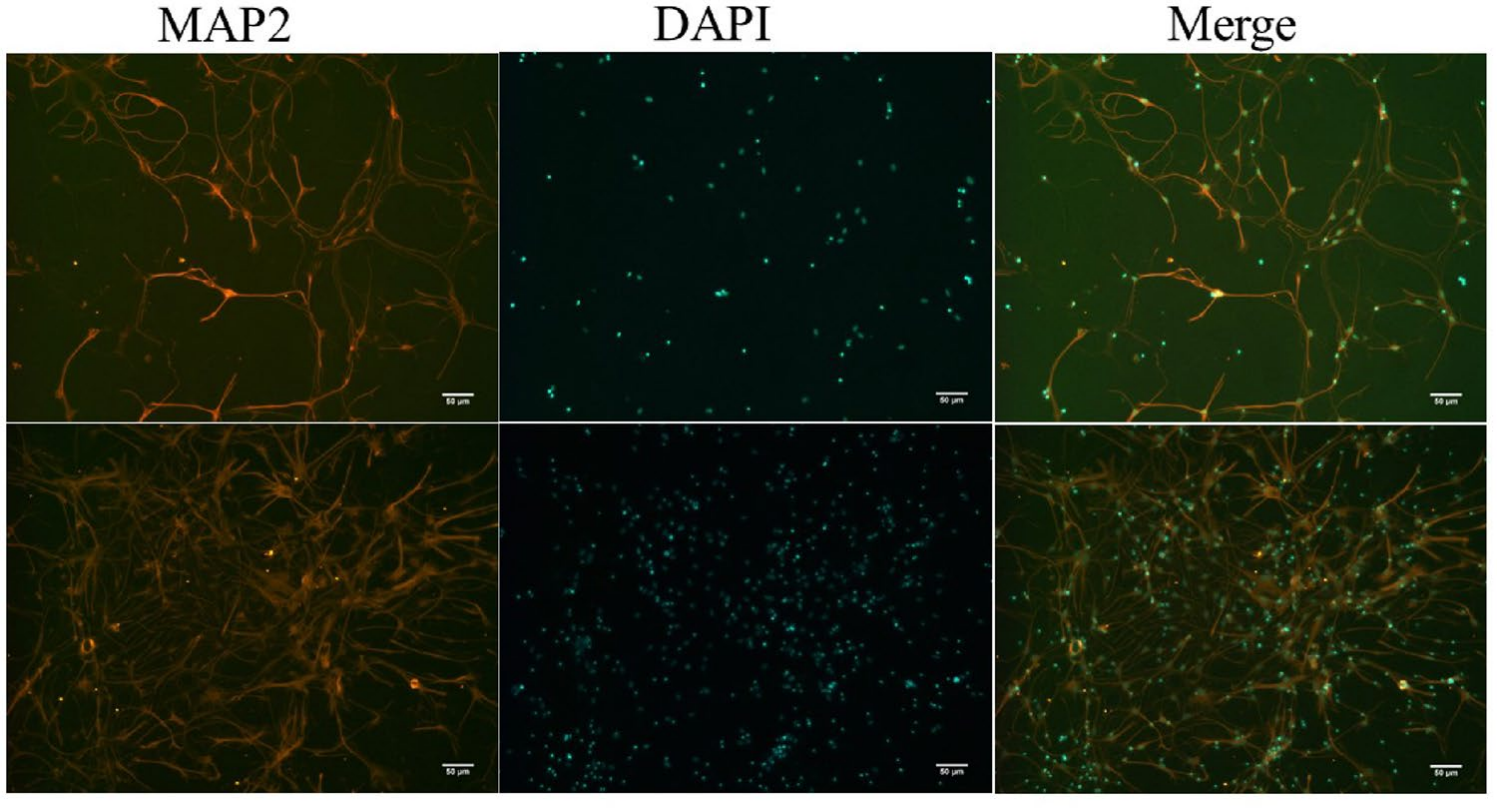
Immunofluorescence staining of rat hippocampal neurons (×200). The cell bodies and processes of hippocampal neurons stained with MAP2 antibody were orange-red, all nuclei stained with DAPI were blue, Merge was a dual fluorescence image after the integration of orange-red MAP2 and blue DAPI images (×200).

As shown in Fig. 1, the cell bodies and protrusions of the primary hippocampal neuronal cells were indicated by staining of MAP2 (orange-red), and the nuclei were stained with DAPI. We determined that approximately 95% of the blue fluorescent particles were surrounded by orange-red fluorescence, indicating that the purity was approximately 95%.

### Hippocampal neuronal hypoxia deprived cell model prepared with CoCl2

**Figure 2:**
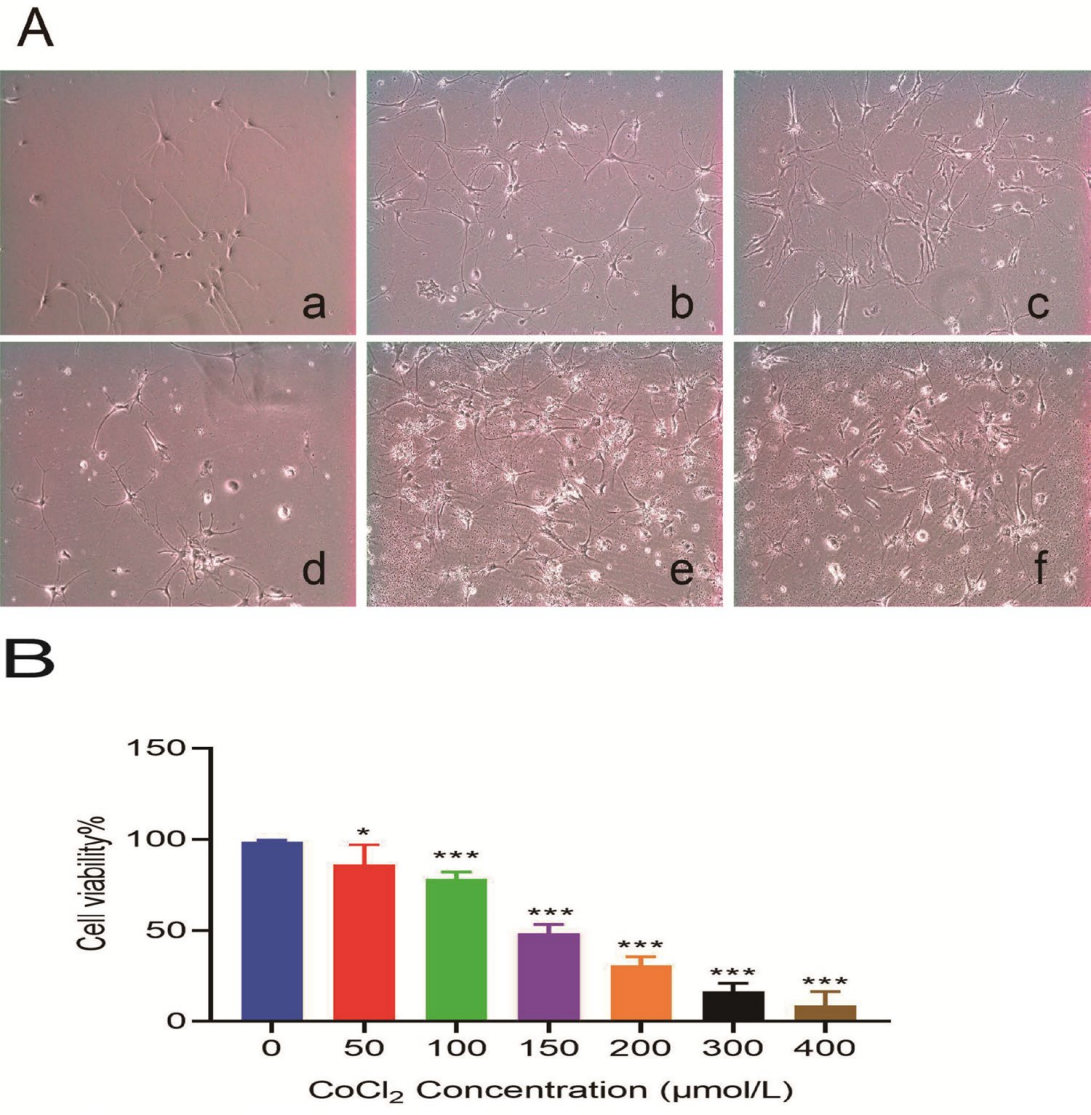
(**A**) Cell morphology of hippocampal neuronal cells treated with different concentrations of CoCl2 for 24 h (× 200) (a, b, c, d, e, f respectively represent 50 μmol/l, 100 μmol/l, 150 μmol/l, 200 μmol/l, 300 μmol/l, 400 μmol/l CoCl2 group). (**B**) Cell viability of hippocampal neuronal cells treated with different concentrations of CoCl2 for 24 h (n = 6 for every group, ^*^P < 0.05, ^***^P < 0.001 vs. normal group by one-way ANOVA).

As shown in Fig. 2A, the hippocampal neuronal cells treated with 50 μmol/l, 100 μmol/l, and 150 μmol/l CoCl2 showed intact cell bodies, clear synapses, fragmented nuclei, and a small portion of necrotic cell debris. In addition, as the concentration of CoCl2 increased from 200 to 400 μmol/l, the cell bodies became smaller or even ruptured, the synapse shortened, the synaptic connection gradually disappeared, and the necrotic cell fragments dramatically increased. Meanwhile, as shown in Fig. 2B, the cell viability of hippocampal neuronal cells decreased in a concentration-dependent manner in corresponding groups. Given that the cell activity of hippocampal neuronal cells is about 50% relative to the control counterpart under 150 μmol/l CoCl2 treatment, we choose the CoCl2 concentration of 150 μmol/l as the optimal concentration for establishing a hypoxic injury model of hippocampal neuronal cells in vitro.

### The appropriate intervention concentration and time of IMP on CoCl2-induced hypoxic hippocampal neuronal cells

**Figure 3:**
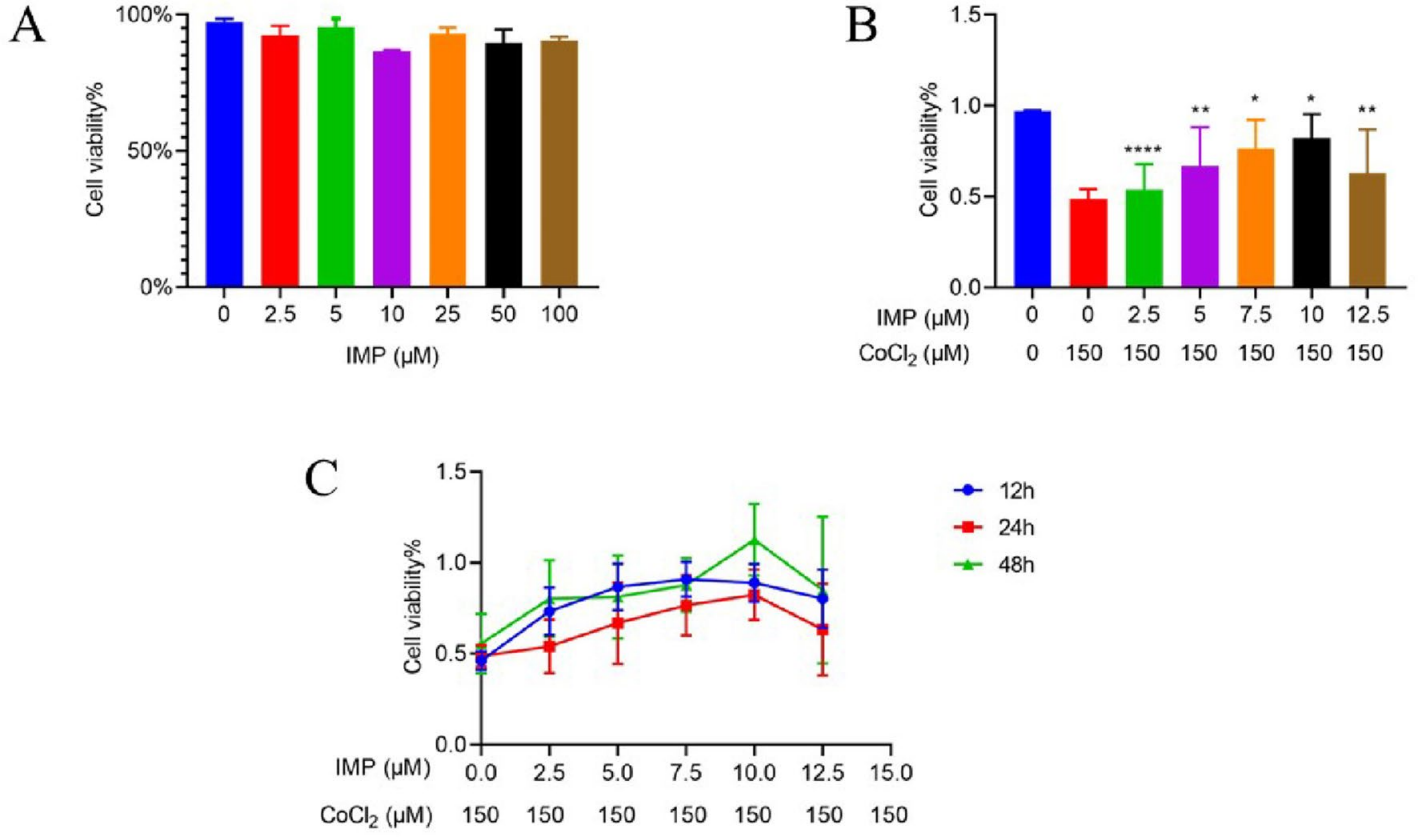
The effects of IMP on the cell viability of CoCl2-induced hypoxia hippocampal neuronal cells. (**A**) Cell viability of normal hippocampal neuronal cells treated with different concentrations of IMP for 24 h. (**B**) Cell viability of CoCl2-induced hypoxia hippocampal neuronal cells treated with different concentrations of IMP for 24 h. (**C**) The interventional time of the viability of CoCl2-induced hypoxia hippocampal neuronal cells. (n = 6 for every group, ^*^P < 0.05, ^**^P < 0.01, ^****^P < 0.0001, vs. CoCl2-induced control group by one-way ANOVA).

As shown in Fig. 3A, different concentrations of IMP (2.5 μmol/l, 5.0 μmol/l, 10 μmol/l, 50 μmol/l, 100 μmol/l) have no significant effect on the activity of normal hippocampal neuronal cells. The cell viability of CoCl2-induced hypoxia hippocampal neurons in the 5.0 μmol/l, 7.5 μmol/l, and 10 μmol/l IMP groups gradually increased with the increase of drug concentration, while the cell viability of hippocampal neurons in the 12.5 μmol/l IMP group began to decrease (Fig. 3B). As shown in Fig. 3C, there was no significant difference in the cell viability of CoCl2-induced hypoxia hypoxia hippocampal neuronal cells of 24 h pretreatment with IMP compared with 12 h and 48 h pretreatment with IMP. Through our experimental results, we finally screened the three concentrations of 5.0 μmol/l, 7.5 μmol/l, and 10 μmol/l as the appropriate interventional concentration of IMP, and the 24 h as the appropriate time for this study.

### IMP prevents apoptosis on the VD cell model

**Figure 4:**
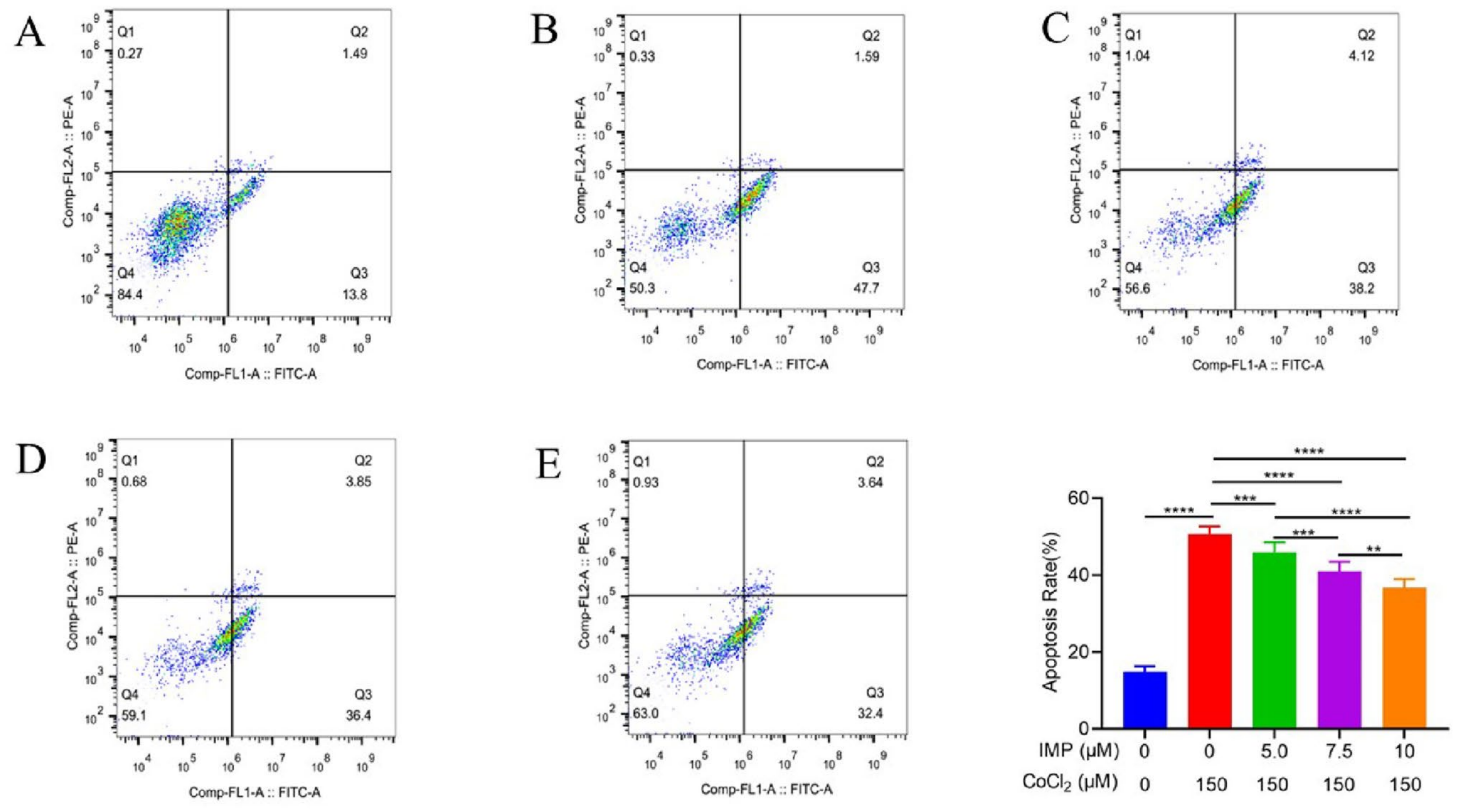
The effect of IMP on the apoptosis rate of CoCl2-induced hypoxia hippocampal neuronal cells. In the figure, the upper left quadrant is dead cells (Q1), the upper right quadrant is late apoptotic cells (Q2), the lower right quadrant is early apoptotic cells (Q3), and the lower left quadrant is living cells (Q4). The total number of apoptotic cells is the sum of Q2 + Q3. (**A**) Normal group, (**B**) CoCl2 treatment group, (**C**) CoCl2 + 5.0 μmol/l IMP, (**D**) CoCl2 + 7.5 μmol/l IMP, (**E**) CoCl2 + 10.0 μmol/l IMP. (n = 6 for every group,, ^**^P < 0.01, ^***^P < 0.01, ^****^P < 0.0001).

As shown in Fig. 4, compared with the normal group, the apoptosis rate of the CoCl2 control group was higher, and the difference was statistically significant (*P* < 0.0001). The apoptosis rate of hippocampal neuronal cells after intervention with 5.0 μmol/l, 7.5 μmol/l, and 10 μmol/l IMP for 24 h was significantly lower than that of the CoCl2 group alone.

### IMP dereases mitochondrial membrane potential on the VD cell model

**Figure 5:**
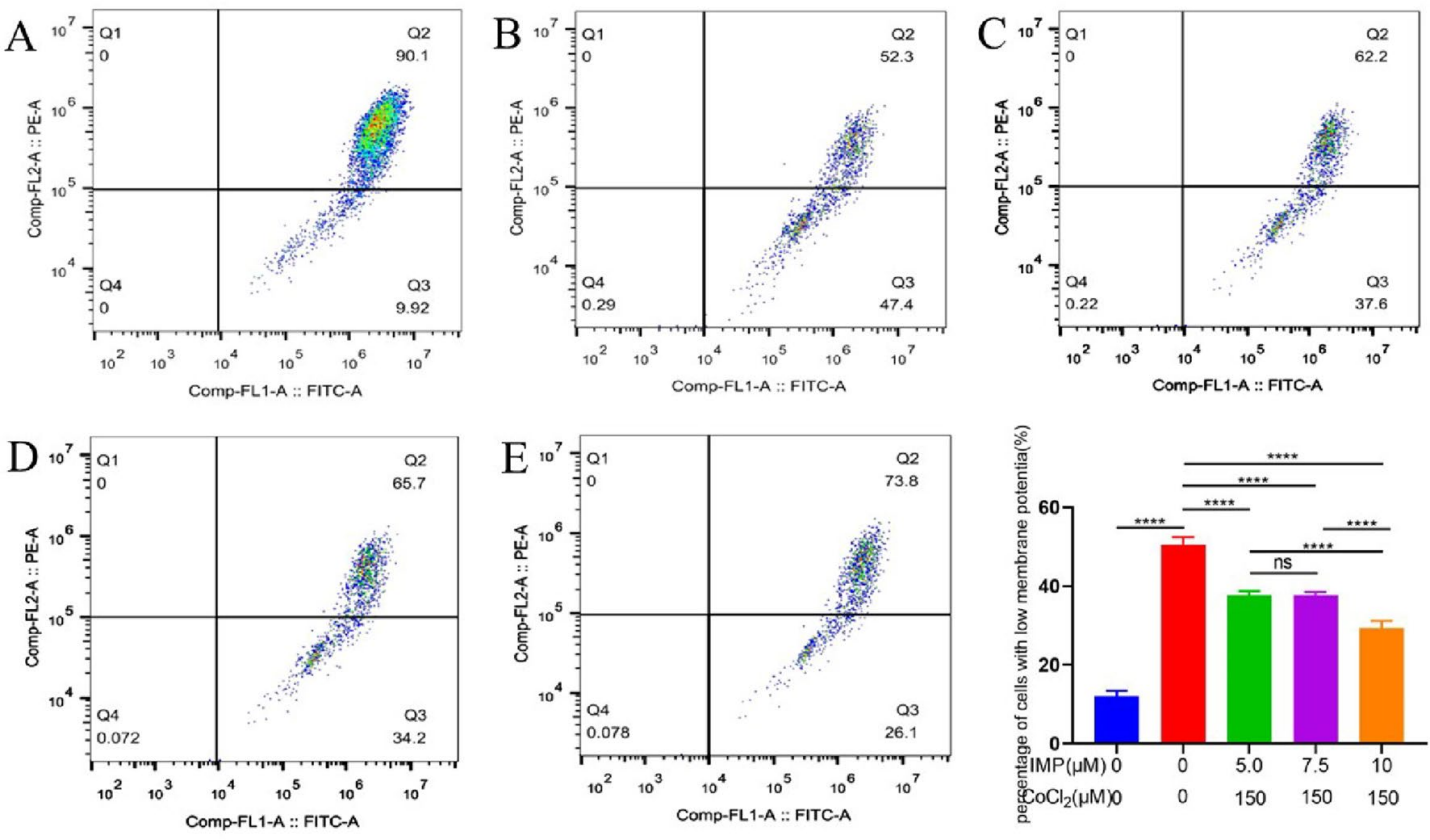
The effect of IMP on the mitochondrial membrane potential of CoCl2-induced hypoxia in hippocampal neuronal cells. The Q2 quadrant in the figure is the polymer of JC-10, which is expressed as red fluorescence. When the mitochondrial membrane potential drops, the Q3 quadrant of JC-10 decomposed into monomers and expressed as green fluorescence. (**A**) Normal group, (**B**) CoCl2 treatment group, (**C**) CoCl2 + 5.0 μmol/l IMP, (**D**) CoCl2 + 7.5 μmol/l IMP, (**E**) CoCl2 + 10.0 μmol/l IMP. (n = 6 for every group; ns, there was no statistical difference; ****P < 0.0001).

As shown in Fig. 5, in the normal group, the intensity of red fluorescence was strong, a small amount of green fluorescence was visible, and the ratio of red fluorescence/green fluorescence was larger. In the control group, the intensity of green fluorescence increased compared to normal group (^**^P < 0.01). After different concentrations of IMP interfered with hippocampal neuronal cells for 24 h, the intensity of green fluorescence gradually weakened compared to control group (^****^P < 0.01). Moreover, there was significant difference in 10 μmol/l IMP group compared to 5.0 μmol/l, 7.5 μmol/l IMP group (^****^P < 0.01). While, there was no statistical difference between 5.0 and 7.5 μmol/l IMP group.

### IMP promotes the nuclear translocation of Nrf2 protein on VD cell model

**Figure 6:**
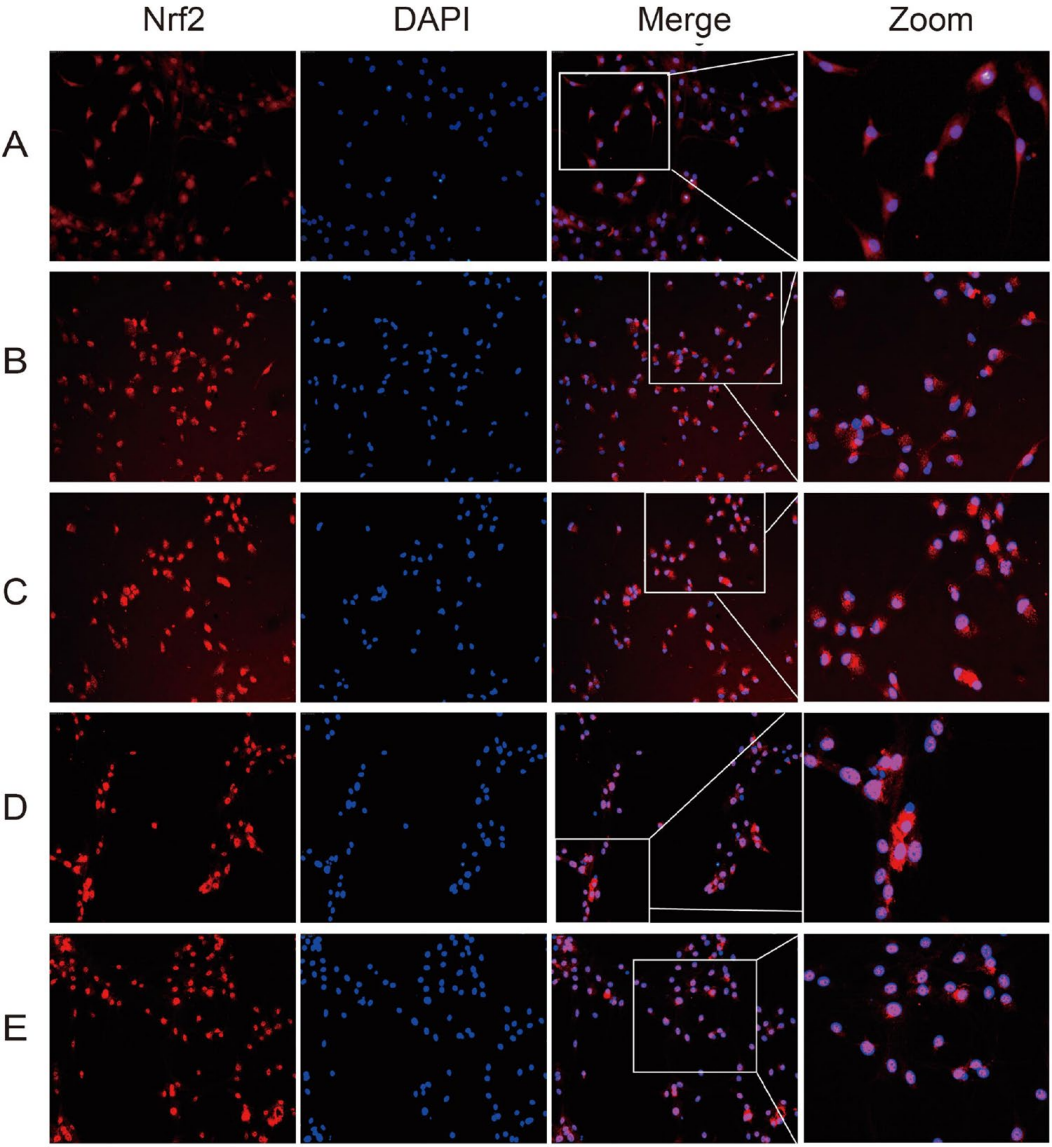
The effect of IMP on the nuclear translocation of Nrf2 protein in CoCl2-induced hypoxia in hippocampal neuronal cells (× 400). Red fluorescence is positive staining for Nrf2 protein, indicating the expression of Nrf2 protein, and blue fluorescence is DAPI staining, indicating cell nucleus. Emerge image is a fusion image of Nrf2 protein and cell nucleus for positioning. The Zoom image is a magnified image randomly selected from the emerge image to observe the Nrf2 positioning. (**A**) Normal group, (**B**) CoCl2 treatment group, (**C**) CoCl2 + 5.0 μmol/l IMP, (**D**) CoCl2 + 7.5 μmol/l IMP, (**E**) CoCl2 + 10.0 μmol/l IMP.

As shown in Fig. 6, under normoxia of hippocampal neuronal cells, Nrf2 protein is mainly located in the cytoplasm (Fig. 6A). While CoCl2 induces the hypoxic state, the red fluorescence of Nrf2 is partly concentrated in the nucleus and partly overlaps with the nucleus (Fig. 6B). In the CoCl2 + 5.0 μmol/l IMP group, more Nrf2 red fluorescence gathered in the nucleus and merged with the nucleus (Fig. 6C). In the CoCl2 + 7.5 μmol/l IMP and CoCl2 + 10 μmol/l IMP groups, the red fluorescence of Nrf2 was completely concentrated in the nucleus and fused with the nucleus (Fig. 6D and E).

### IMP plays antioxidative effects by increasing the mRNA and protein expression levels of Nrf2, NQO-1, and HO-1 on VD cell model

**Figure 7:**
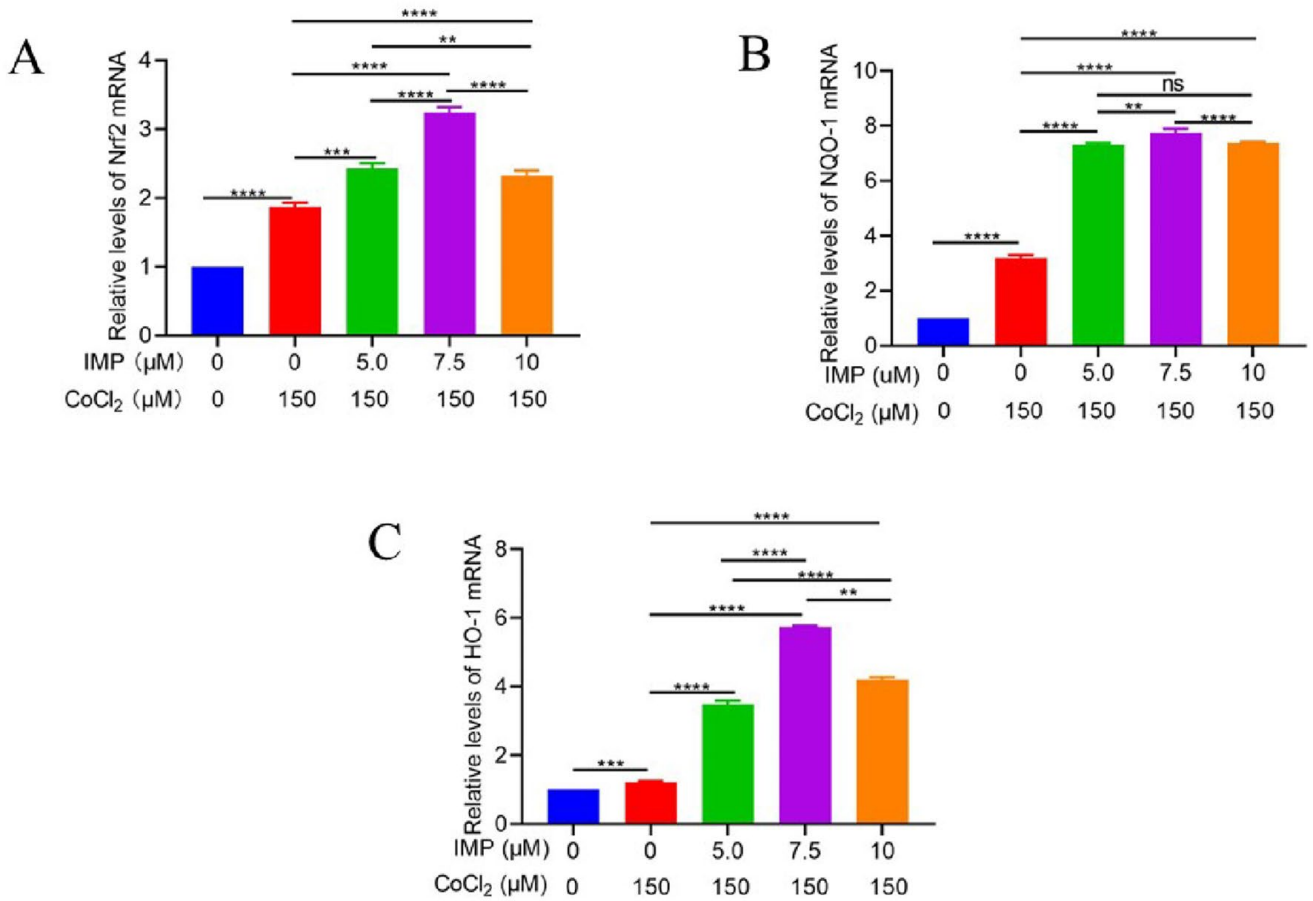
The effects of IMP on the expression of Nrf2, NQO-1, HO-1 mRNA after 24 h intervention in hippocampal neuronal cells. (**A**) Nrf2 mRNA, (**B**) NQO-1 mRNA, (**C**) HO-1 mRNA. (n = 6 for every group, ns, there was no statistical difference, ^**^P < 0.01, ^***^P < 0.001, ^****^P < 0.0001).

**Figure 8:**
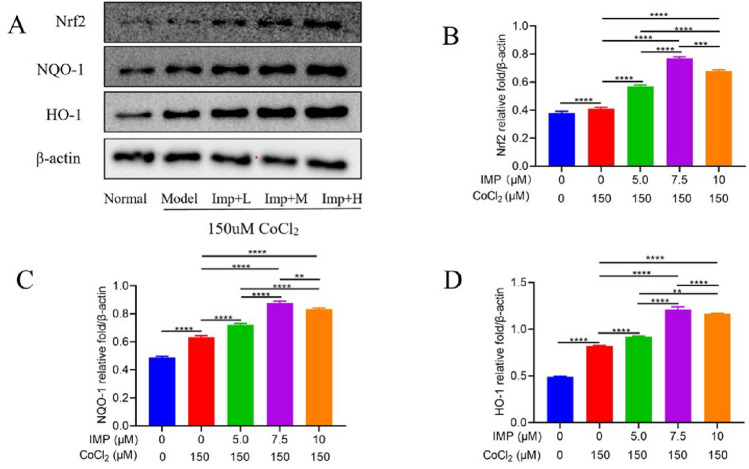
The effects of IMP on the expression of Nrf2, NQO-1 and HO-1 protein after 24 h intervention in hippocampal neuronal cells. (**A**): the western blot images of Nrf2, NQO-1, HO-1, and β-actin expression, (**B**): the relative expression of Nrf2, (**C**): the relative expression of NQO-1, (**D**): the relative expression of HO-1. Dividing lines were used to make explicit for the grouping of blots cropped from diferent parts of the same gel or from diferent gels. The experiment was repeated three times, and the data was shown as mean ± standard deviation. (n = 3 for every group, ^**^P < 0.01, ^***^P < 0.001, ^****^P < 0.0001).

As shown in Figs. 7 and 8, RT-PCR and Western blot analysis showed that compared with the control group, different concentrations of IMP could up regulate the mRNA and protein levels of Nrf2, HO-1 and NQO-1. Furthermore, 7.5 μmol/l IMP group had the most significant effect among three IMP groups. These changes showed that IMP could protect neurons through Nrf2 pathway.

### Imperatorin exerts an antioxidant effect on the VD cell model by regulating the Nrf2 signaling pathway

**Figure 9:**
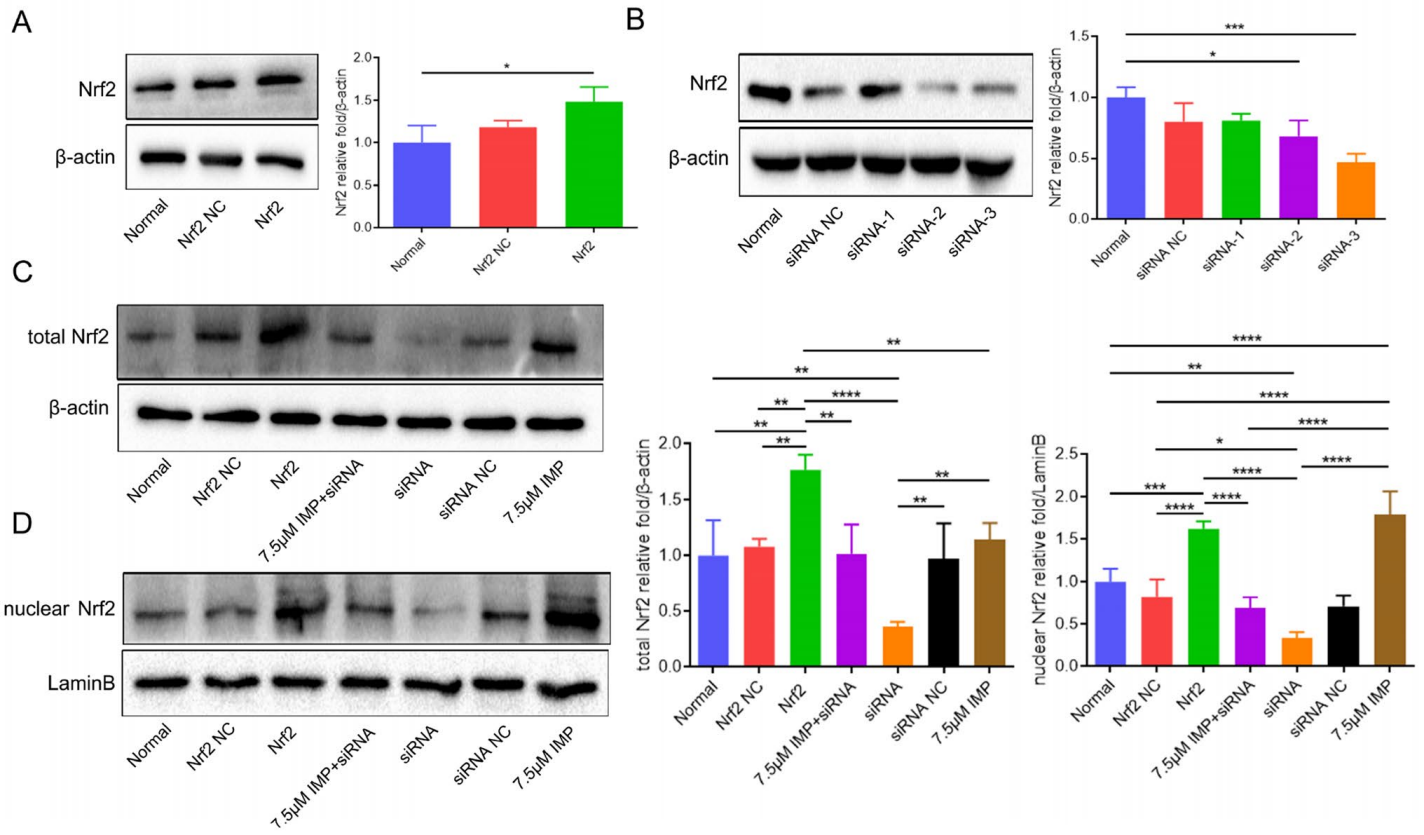
IMP exerts a protective effect on the VD cell model prepared by CoCl2-induced hypoxic hippocampal neuronal cells by regulating the Nrf2 signaling pathway. (**A**): the relative expression of Nrf2 after transfection of the overexpression vector. (**B**): the relative expression of Nrf2 after siRNA-1, siRNA-2, siRNA-3 transfection. (**C**): relative expression of total Nrf2 in VD cell model by different treatments. (**D**): relative expression of nuclear Nrf2 in VD cell model with different treatments. Dividing lines were used to make explicit for the grouping of blots cropped from diferent parts of the same gel or from diferent gels. The experiment was repeated three times, and the data was shown as mean ± standard deviation. (n = 3 for every group, ^*^P < 0.05, ^**^P < 0.01, ^***^P < 0.001, ^****^P < 0.0001 by one-way ANOVA).

As shown in Fig. 9A and B, the cell transfection test found that compared with the normal group, the total protein expression level of Nrf2 in the Nrf2 overexpression group was significantly increased, indicating that the Nrf2 overexpression vector effectively enhanced the expression of the Nrf2 gene. In addition, the transfection effect was verified by western blot, and the sequence with the best interference effect was screened out. The Nrf2 protein expression level of the Nrf2-siRNA3 transfection group was significantly lower than that of the other four groups of cells. Therefore, we selected the Nrf2-siRNA3 transfection group for subsequent experiments (^***^P < 0.001). As shown in Fig. 9C and D, compared with the normal group, the total Nrf2 and nuclear Nrf2 proteins in the Nrf2 overexpression group were significantly increased, and the Nrf2-siRNA3 transfection group was significantly decreased in the Nrf2 protein expression level, which further verified our successful transfection effect. In addition, we found that compared with the normal group, the IMP single group had no significant effect on the expression of total Nrf2 protein in cells, but significantly increased the level of Nrf2 protein in the nucleus (^***^P < 0.0001), indicating that IMP can facilitate nuclear translocation of Nrf2. Compared with the Nrf2 siRNA group, total Nrf2 and nuclear Nrf2 proteins were significantly increased in the IMP group (^*^P < 0.01, ^**^P < 0.0001, respectively). Compared with the IMP single group, the nuclear Nrf2 protein in the IMP + Nrf2 siRNA group was significantly decreased, indicating that transfection of siRNA could completely reverse the effect of IMP on activating the Nrf2 signaling pathway(^****^P < 0.0001).

**Figure 10:**
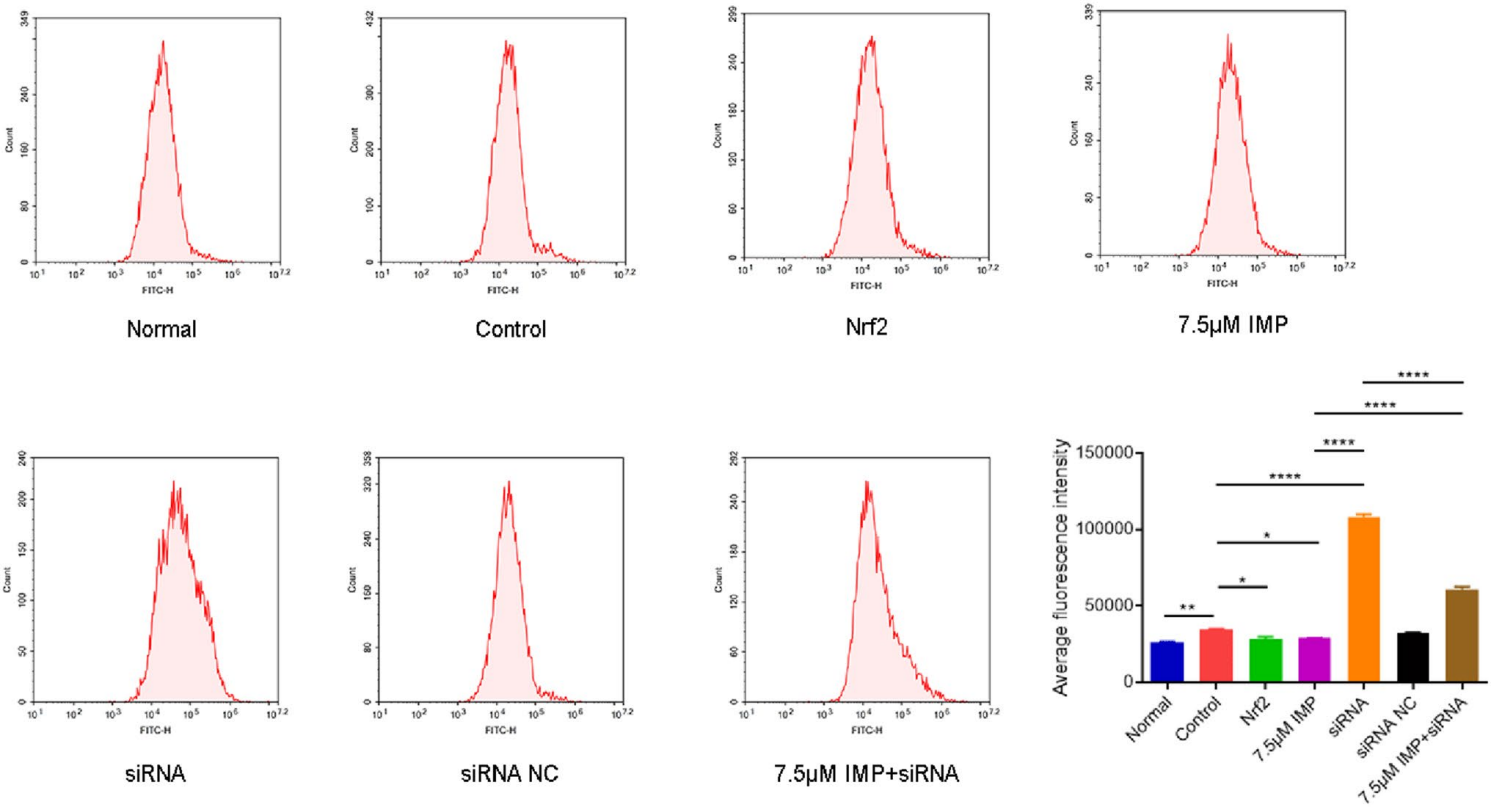
Effects of different treatments on ROS content in VD cell model. The experiment was repeated three times, and the data was shown as mean ± standard deviation. (n = 3 for every group, ^*^P < 0.05, ^**^P < 0.01, ^****^P < 0.001 by one-way ANOVA).

The results were shown in Fig. 10. Compared with the normal group, oxidative stress occurred in hippocampal neuronal cells after CoCl2 treatment for 24 h, and the level of ROS increased significantly (^**^P < 0.01). Compared with the cells treated with CoCl2 alone, the ROS level of hippocampal neuronal cells in the Nrf2 siRNA group was further increased, with statistically significant differences (^****^P < 0.0001). ROS levels in hippocampal neuronal cells were significantly lower in the Nrf2 overexpression group (^*^P < 0.05). In addition, compared with the control group, the level of ROS in hippocampal neuronal cells in the IMP treatment group decreased significantly (^*^P < 0.05). Compared with the IMP treatment group, the Nrf2 siRNA group showed a significant increase in the level of ROS (^****^P < 0.0001).

## Discussion

In the present study, we demonstrated that IMP can reduce oxidative stress damage in CoCl2-induced hypoxic VD cell model. The underlying pharmacological mechanism may be directly through its anti-oxidative and anti-apoptosis effects, and was related to the regulation Nrf2 antioxidative pathway.

As an important component of the traditional Chinese medicine Angelica dahurica, IMP has been studied in bronchial asthma^19^, tumors, cardiovascular diseases, etc^20,21^, but researches in neurology are still lacking. Previous studies have found that it has pharmacological effects such as anti-oxidative stress, anti-inflammatory, anti-apoptosis, blocking calcium channels, and anti-convulsions^22^. Meanwhile, the key pathological mechanism of VD is the complex pathological processes such as free radicals, oxidative stress, inflammation and neuronal apoptosis caused by chronic hypoperfusion injury. And our previous studies have found that IMP can up-regulate the expression of Bcl-2 protein, decrease the expression of Bax and Caspase-3 protein, reduce cell apoptosis, and improve the learning and memory ability of VD model rats^18^. In this study, in order to further explore whether IMP has direct anti-apoptosis and anti-oxidative effects, we bulit VD cell model by CoCl2-induced hypoxia in hippocampal neuronal cells in vitro, which is internationally recognized and confirmed^23^.

Mitochondria plays a central role in many cellular processes, such as ATP production, apoptosis and β-oxidation of fatty acids, which is thought to be responsible for the production of free radicals and is also targets of oxidative stress damage^24^. It has been reported that the change of mitochondrial membrane potential is a milestone event of apoptosis. It occurs prior to typical apoptotic features such as nucleus pyknosis and DNA fragmentation^25^. Ischemia and hypoxia injury can lead to a decreas in mitochondrial membrane potential, which can activate the classical apoptosis signaling pathway of caspase-3, resulting in a series of hierarchical reactions, and that ultimately leading to apoptosis^26^. In this study, it is found that the apoptosis rate was increasing, and mitochondrial membrane potential was reducing, resulting in the cell membrane swelling and rupture in hippocampal neuronal cells of CoCl2-induced chemical hypoxia, which is in line with previous studies^27^. Moreover, the apoptosis rate was decreased, mitochondrial membrane potential rise obviously after interference with IMP, which indicating that IMP has a neuroprotective effect on hippocampal neuronal cells. The neuroprotective mechanism of IMP may be the preventing mitochondria from damage and avoiding the activation of apoptotic pathways.

Oxidative stress plays a vital role in neuronal damage after ischemia. Free radicals generated during ischemia far exceed the scavenging ability of its own endogenous antioxidant system, which can not only directly damage cells and lead to cell necrosis, but also indirectly lead to cell apoptosis through mitochondrial pathways, DNA repair enzymes and transcription factors^28,29^. It has been shown that transcription mediated by Nrf2 activates a variety of antioxidantive genes and second-stage detoxification enzyme genes, thereby reducing ROS and electrophilic substances caused by cell damage and maintaining the physiological balance of oxidation-antioxidant^11^. Nrf2 binds to and stabilizes Keap1 in the cytoplasm in normoxic state. Under hypoxia, Keap1 is isolated from Nrf2 protein, which enters the nucleus and binds to the antioxidant reaction element ARE to induce the expression of a series of antioxidant enzymes. Activated of Nrf2/ARE signaling pathway plays an antioxidant effect^30^.

Several studies have determinated that traditional Chinese medicine have neuroprotective effects by activating Nrf2/ARE signaling pathway^31,32^. Such as, Yang C et al^33^ found that curcumin could protect neuronal cells by activating the Nrf2/ARE signaling pathway in the study of cerebral infarction. Studies by Li and Chen et al^34,35^ also confirmed that ursolic acid could also promote the recovery of nervous system by activating the Nrf2/ARE signaling pathway. It has been confirmed that tert-butylhydroquinone, a specific activator of Nrf2/ARE signaling pathway, can exert neuroprotective effects through Nrf2/ARE signaling pathway in various central nervous system diseases^36^. In our experiment, we observed the location of Nrf2 protein by laser confocal microscope, and found that Nrf2 protein exists in the cytoplasm of hippocampal neuronal cells under normoxia state, while neuronal cells undergo oxidative stress under hypoxia state after CoCl2 treatment. These results were consistent with previous studies^37^. In addition, our results showed that Nrf2 protein enters the nucleus, and the level of ROS increases significantly, which is similar with some researches^38^. After 24 h intervention of IMP, hippocampal neurons were fused with nucleus completely. Meanwhile, IMP can up-regulate the expression of Nrf2, HO-1, NQO-1 mRNAs and proteins, significantly increase the level of Nrf2 protein, and reduce the level of ROS in hippocampal neuronal cells, suggesting that IMP may activate Nrf2 signaling pathway, and have an antioxidant effect on CoCl2-induced hypoxic hippocampal neuronal cells. Our findings were similar to the results reported by Aarti et al. that resveratrol attenuated mitochondrial oxidative stress in Vascular dementia through Nrf2 signaling^39^. Accordingly, the effects of the up-regulating HO-1 protein expression and the reducing the level of ROS by IMP were also completely blocked by Nrf2 siRNA. These results indicated that overexpression of Nrf2 can promote the antioxidant activity of IMP, while transfection of Nrf2 siRNA can completely reverse the antioxidant effect of IMP, suggesting that IMP can prevent oxidative stress injury of hypoxia hippocampal neurons induced by CoCl2 by regulating Nrf2 pathway.

**Figure 11:**
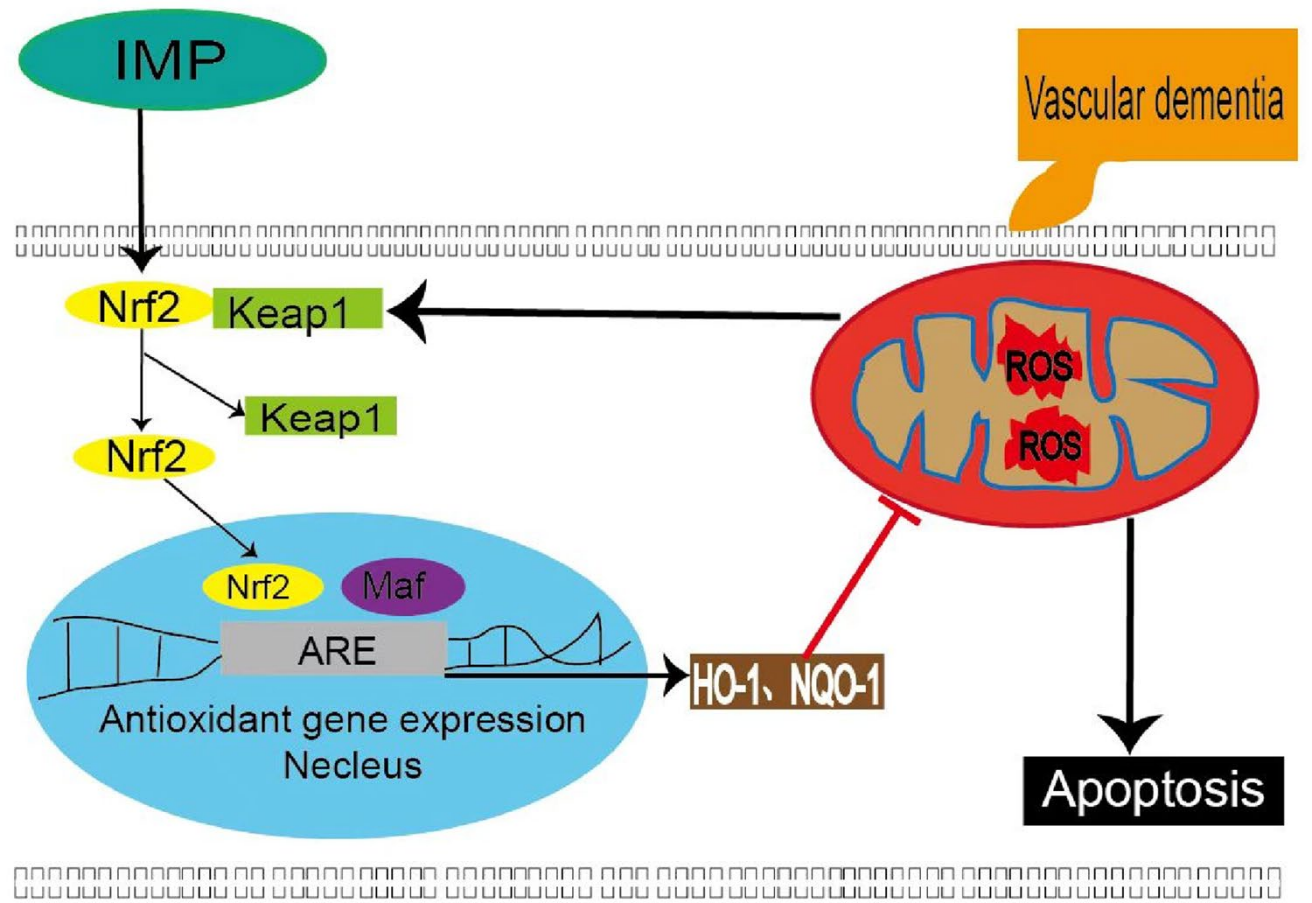
Schematic diagram of the antioxidative effect of imperatorin by regulating the Nrf2 signaling pathway.

There are several limitations in this study. First, the subjects selected in this experiment were limited to hippocampal neuronal cells, therefore, cerebral cortex cells or other neuronal cells could be further studied. Meanwhile, in the complex pathological mechanism of VD, in addition to oxidative stress, there is also immune inflammatory response. And based on the known pharmacological effects of IMP, IMP may not only act through anti-oxidative stress and anti-apoptosis, but also play a neuroprotective role through other pharmacological effects such as anti-inflammatory and inhibition of cholinease activity. Furthemore, it is unclear that the specific mechanism of Nrf2 entered the nucleus and combined to the neuclear antioxidant element ARE under hypoxia state. After Nrf2 enters into the nucleus, there may be other processes such as phosphorylation degradation and ubiquitination modification. Whether it may be affected by other proteins during this process is still unclear, and further research is needed (Fig. 11).

## Conclusion

We demonstrated that IMP can increase the mitochondrial membrane potential of hippocampal neuron, reduce ROS level, reduce oxidative stress damage, inhibit hippocampal neuroapoptosis, and ultimately reduce the oxidative stress damage in the VD cell model prepared by CoCl2 induced hypoxia in hippocampal neurons. On the other hand, the antioxidant activity of IMP can be completely blocked by Nrf2 siRNA transfection, while Nrf2 overexpression can completely reverse the antioxidant effect of IMP. This suggests that IMP can prevent CoCl2-induced hypoxic hippocampal neurons from oxidative stress damage by regulating the Nrf2/ARE pathway. These findings provide a new perspective on the progression of VD disease and prove that IMP is a promising neuroprotective agent.

## Supplementary Information

Supplementary Information.

## Data Availability

The raw data supporting the conclusions of this article will be made available by the authors. Requests to access the datasets should be directed to huangying0202@126.com.
