# Supplementary material for: Imperatorin exerts antioxidant effects in vascular dementia via the Nrf2 signaling pathway
Source: Sci Rep. 2023 Apr 5;13:5595. doi: 10.1038/s41598-022-21298-x (PMC10076271; doi:10.1038/s41598-022-21298-x)
Supplement: Supplementary file 1 — Supplementary Information. [file 41598_2022_21298_MOESM1_ESM.pdf]

Figure 8. Example of original western blot for three repeats

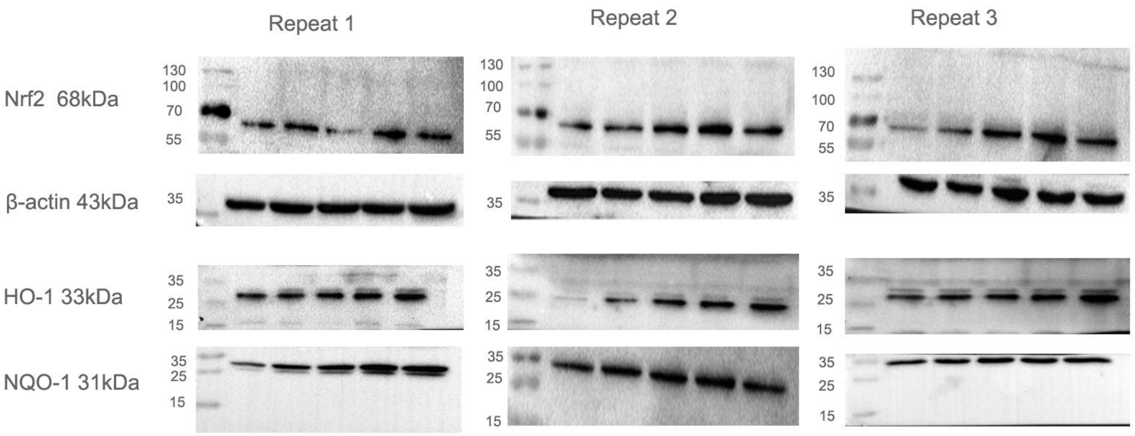

Figure 9. Example of original western blot for three repeats

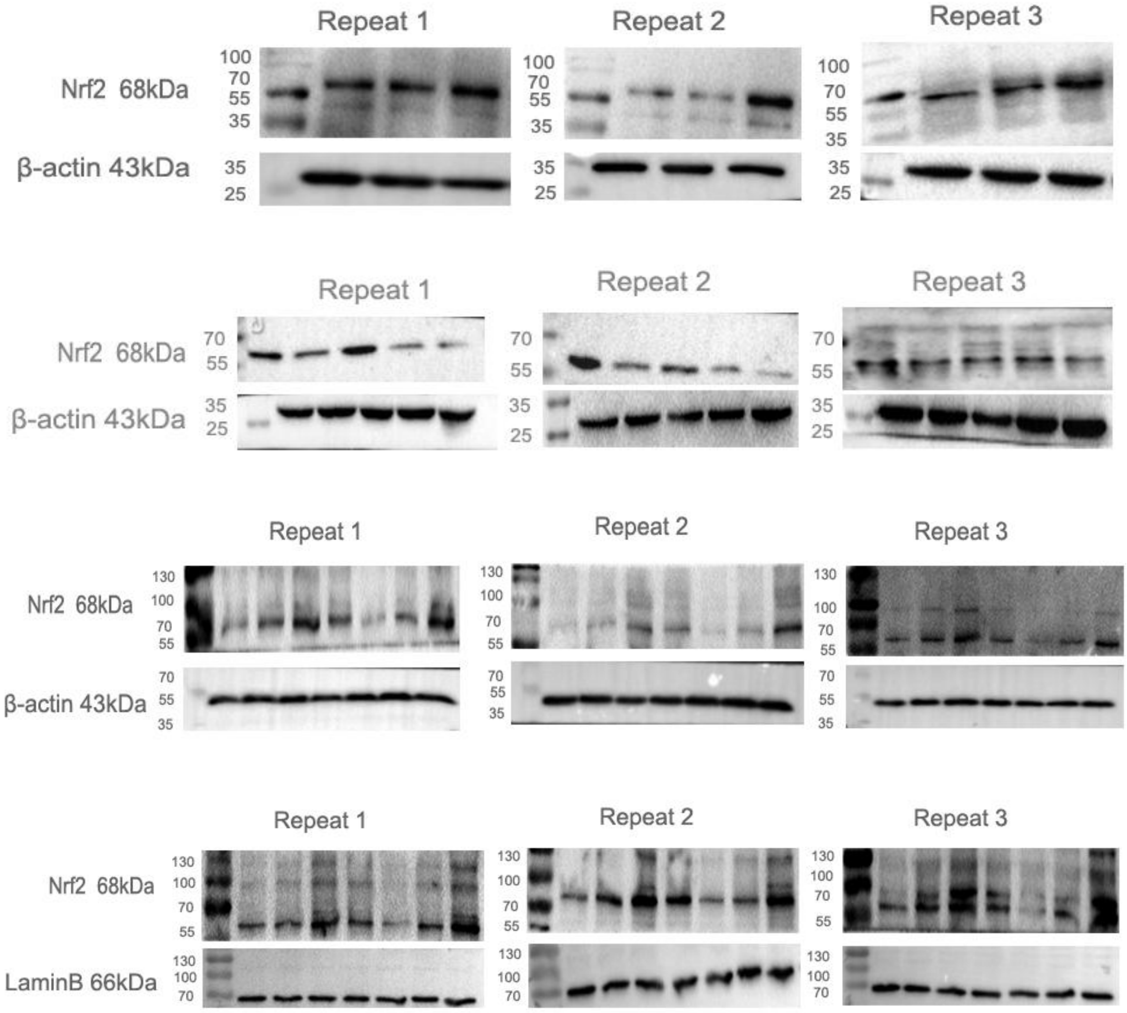

If the whole membrane was cut to blot for different antibodies, you are required to load all pieces of whole blot and the molecular marker must be shown in each blot. Figure 8 and Figure9 showed the whole blot after cutting membrane at molecular weight 70 kDa, 55 kDa, 35 kDa and 15~25 kDa for Nrf2 (68 kDa),LaminB(66 kDa), actin (43 kDa),HO-1 (33 kDa) and NQO-1 (31 kDa).
